# Analysis of factors affecting the tip position of a tunneled cuffed catheter using the thoracic surface marker method

**DOI:** 10.1080/0886022X.2025.2496435

**Published:** 2025-05-06

**Authors:** Ce Liu, Xiaojing Zhao, Huanhuan Wang, Yan Huang, Sijia Ma, Zhangyuan Shi, Shuzhong Duan

**Affiliations:** a Nephrology Department, The Affiliated Hospital of Chengde Medical University, Chengde, China; b Metabolic Vascular Disease Group, Hebei Key Laboratory of Panvascular Diseases, Chengde, China

**Keywords:** Tunneled cuffed catheter, cardiothoracic ratio, thoracic diameter, thoracic aspect ratio, sternal length, thoracic surface marker

## Abstract

Catheterization methods use the lower margin of the right third intercostal space to predict the catheter tip position; however, adjustments are often necessary. We evaluated the catheter position and surface markers of the chest and the relevant factors that affect the position and depth of the catheter tip. This single-center, cross-sectional study included 173 patients who underwent right internal jugular vein tunnel insertion with a tunneled cuffed catheter (TCC). The sternal length, intercostal width, anterior-to-posterior sternal diameter, and transverse diameter of the thorax were recorded, and the thoracic aspect ratio was calculated. The cardiothoracic ratio and position of the catheter tip were measured using chest computed tomography. The correct placement was when the catheter tip was in the upper 1/3 of the right atrium (deep group: below the correct placement; shallow group: above the correct placement). Catheter tip indices were subjected to logistic regression analyses. A receiver operating characteristic (ROC) curve was used to assess the risk and optimal cutoff of the catheter tip depth. There were significant differences in sternum length, cardiothoracic ratio, anterior-to-posterior thoracic diameter, and thoracic aspect ratio between the groups (*p* < 0.05). The latter three variables were influencing factors for catheter tip depth (*p* < 0.05). The anterior-to-posterior thoracic diameter (*p* = 0.003; cutoff, 22.5 cm; ROC, 0.695) and thoracic aspect ratio (*p* = 0.014; cutoff, 0.74; ROC, 0.632) were independent risk factors for catheter tip depth and position, respectively. The position of the TCC catheter tip was related to the sternal length, anterior-to-posterior thoracic diameter, thoracic aspect ratio, and cardiothoracic ratio.

## Introduction

Most patients with end-stage renal syndrome choose hemodialysis as the mode of dialysis treatment [1]. Although hemodialysis access experts adhere to the principle of internal fistula priority, due to the influence of multiple factors, such as old age, diabetes, hypertension, and hyperlipidemia, the vascular condition of patients with chronic kidney disease is poor, and the arteriovenous fistula patency rate is low. Therefore, tunneled cuffed catheters (TCCs) remain the most commonly used form of hemodialysis access for patients undergoing dialysis.

In general, the right internal jugular vein is the preferred site for TCC placement. To ensure high enough hemodialysis blood flow and minimize the risk of catheter-related complications, studies suggest placing the catheter tip in the right atrium [2,3], which helps prevent catheter-related complications, such as catheter dysfunction, thrombosis, fibrin sheath formation, and arrhythmia [4–6]. Advanced techniques, such as preoperative chest radiography, intracardiac electrocardiography (ECG), and transesophageal echocardiography (TEE), are recommended in clinical practice in order to place the TCC tip in the correct position. However, ECG and TEE are not commonly used in clinical practice and are time-consuming and expensive [7]. Body surface markers, such as sternal length, intercostal width, and thoracic thickness, are easily identifiable and measurable and can be used with most patients. A soft ruler is generally used to measure the puncture point and length of the third inferior intercostal margin on the right side of the sternum to select the appropriate type of catheter and predict the length of its insertion. The catheterization process is simple; however, errors may occur, and sometimes, the catheter needs to be adjusted accordingly. The purpose of this study was to explore the factors affecting the position of the catheter tip and improve the accuracy of the positioning of the catheter tip, with the lower margin of the third intercostal space on the right side of the sternum as a projection of the right atrium to predict the appropriate positioning of the catheter tip.

## Materials and methods

### Ethical approval

This study was performed in accordance with the Declaration of Helsinki and was approved by the Ethics Committee of the Affiliated Hospital of Chengde Medical University (Ethical approval ID: CYFYLL2022609). All enrolled patients signed an informed consent form.

### Study design and population

This prospective study included patients who underwent TCC for the first time at our hospital between November 2022 and October 2023. These patients have not undergone hemodialysis treatment in the past, and some patients have undergone arteriovenous fistula surgery, but it was unsuccessful or poorly matured. The inclusion criteria were as follows: (1) aged ≥18 years, (2) patients with end-stage renal disease who underwent right internal jugular vein TCC implantation for the first time, (3) provided complete clinical data, and (4) signed the informed consent form. The exclusion criteria were as follows: (1) patients in critical condition who could not tolerate the placement tube, (2) patients with pacemakers, (3) patients with recent infections, (4) patients with thoracic deformity whose body surface markers could not be measured, (5) patients who did not undergo chest computed tomography (CT) after catheterization, and (6) patients with cardiovascular disease or venous vascular diseases. Before surgery, all patients were confirmed to have normal coagulation function, a platelet count above 80 × 10^9^/L, a hemoglobin level of 80 g/L or above, blood pressure controlled below 160/90 mmHg, normal blood oxygen saturation, and no indication of ventricular arrhythmia on electrocardiogram.

### Clinical data collection

The general clinical data, including age, sex, height, weight, and body mass index (BMI), were collected. Sternal length and the second and third intercostal widths of each patient were measured. The thoracocardiac ratio was measured on anterolateral CT images. The anterior-to-posterior and transverse thoracic diameters were measured at the sternal angle, and their ratios were calculated (thoracic aspect ratio).

### Operation

**Figure 1.**
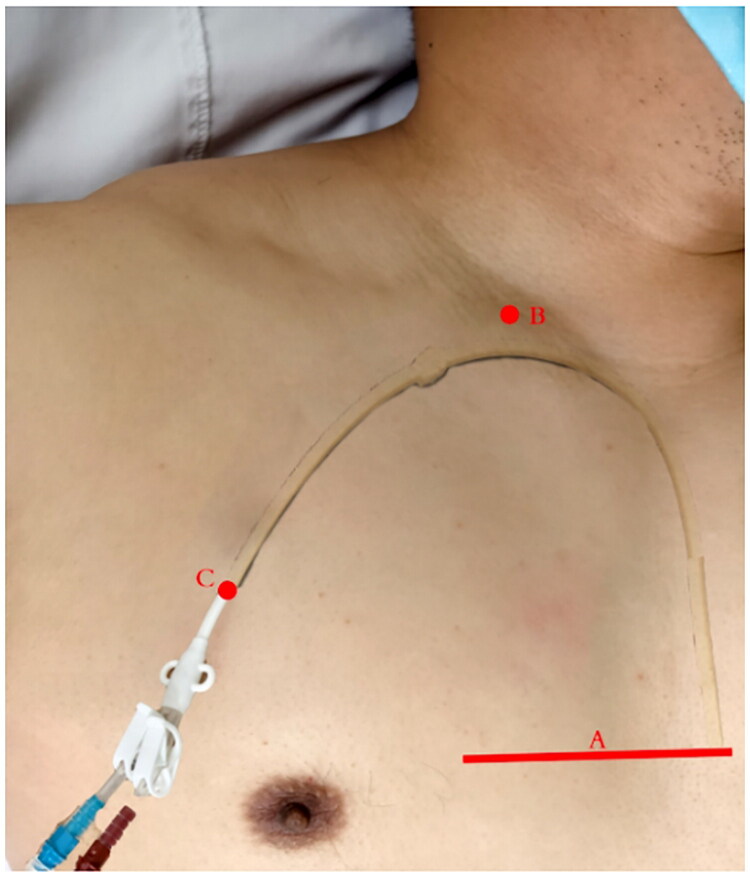
Line a refers to the inferior margin of the right third intercostal space at the sternum. Point B refers to the puncture plane. Point C refers to the catheter outlet location.

The patient was placed in a supine position facing the left side, disinfected with iodophor, and covered with a sterile hole towel. After local infiltration of anesthesia with 1% lidocaine, the internal jugular vein was cannulated under ultrasonographic guidance. Dark red blood reflux was observed to ascertain proper placement. The guidewire was inserted into jugular vein, and the cannulating needle was removed (Figure 1 point B). The body surface projection of the catheter tip was shown at the right parasternal inferior third of the intercostal margin (Figure 1, point A). Based on the distance between points A and B, a catheter of an appropriate length was selected, and the tunnel exit position was determined according to the length of the catheter (Figure 1, point C). Local anesthesia was administered along the subcutaneous tunnel. Transverse incisions approximately 1 cm in length were made at points B and C. The catheter was introduced into the subcutaneous tunnel using a tunneling needle from point C to point B. The cuff of the catheter was located subcutaneously approximately 2–3 cm from point C at the exit. The catheter was placed in the blood vessel along the guidewire. A syringe was used to aspirate and repeatedly inject blood into the arterial and venous ends of the catheter to ensure smooth function. Heparin saline (10 mg/mL) was used to seal the arteriovenous end of the isovolumetric tube, which was then clamped. The heparin cap was then screwed. The incisions at the neck and catheter outlet were closed, the catheter was fixed with sutures, and the incisions were covered with a sterile dressing.

### Determining the position of the catheter tip

**Figure 2.**
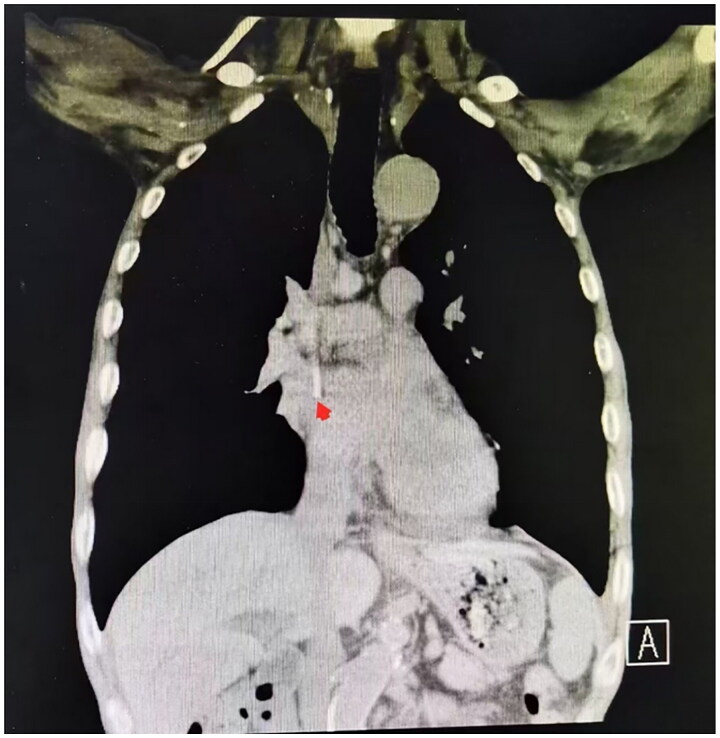
The red arrow indicates the correct tip positioning of the catheter.

A chest CT was performed to determine the location of the catheter tip. The advantage of CT is that it can accurately locate the right atrial target position and the corresponding costal cartilage or intercostal space. After catheter insertion, a chest CT was performed to determine the position of the catheter tip (Figure 2). When the catheter tip was in the upper 1/3 of the right atrium, it was considered to be in the correct placement (correct group). When the catheter tip was below or above the upper 1/3 of the right atrium, it was considered to be too deep or too shallow, respectively (deep and shallow groups, respectively).

### Statistical analyses

The data were analyzed using SPSS 25.0. The measurement data were tested for normality by K-S, and those that met a normal distribution were expressed as mean ± standard deviation. An independent sample *t*-test was used to compare two groups, and multigroup comparisons were performed using one-way analysis of variance. Non-normally distributed data were expressed as median and quartile spacings, and the rank sum test was used. Count data were expressed as rates or component ratios, and comparisons between groups were performed using Fisher test or chi-square tests. Single-factor and multifactor logistic regressions were used to analyze the related factors affecting catheter tip position and depth. The risk of the catheter tip being too deep was assessed using a receiver operating characteristic (ROC) curve. Statistical significance was set at *p* < 0.05.

## Results

### Comparison of patient demographics

A total of 173 patients (77 males and 96 females) with end-stage renal disease who underwent right internal jugular vein TCC for the first time participated in this study. One-hundred eleven patients were in the correct group, 32 were in the deep group, and 30 were in the shallow group; the average ages were 59.42 ± 16.37 years, 54.50 ± 15.75 years, and 63.13 ± 16.20 years, respectively. Chronic glomerulonephritis was the primary cause of primary renal disease in all three groups, followed by diabetic nephropathy. There were no significant differences in sex, age, tube type, or diabetes status among the three groups (*p* > 0.05; Table 1).

**Table 1.** Patient demographics and catheter tip position among the three groups.

	Deep group	Correct group	Shallow group	*P*
Sex				0.843
Male	14 (43.8%)	51 (45.9%)	12 (40%)	
Female	18 (56.2%)	60 (54.1%)	18 (60%)	
Age, years	54.50 ± 15.75	59.42 ± 16.37	63.13 ± 16.20	0.111
Length of catheter				0.138
36 cm	18 (56.3%)	75 (67.6%)	24 (80%)	
40 cm	14 (43.7%)	36 (32.4%)	6 (20%)	
Primary disease				0.308
CGN	20 (62.5%)	75 (67.6%)	18 (60%)	
DM	8 (25%)	23 (20.7%)	4 (13.3%)	
Others	4 (12.5%)	13 (11.7%)	8 (26.7%)	
Comorbidity				
Hypertension				0.040
Yes	16 (50%)	57 (51.4%)	22 (73.3%)	
No	16 (50%)	54 (48.6%)	8 (26.7%)	
DM				0.211
Yes	18 (56.3%)	75 (67.6%)	24 (80%)	
No	14 (43.7%)	36 (32.4%)	6 (20%)	

CGN: chronic glomerulonephritis; DM: diabetes mellitus.

### Comparison of anatomical data

Height, weight, BMI, width of the intercostal space (L2, L3), sternum length, cardiothoracic ratio, anterior-to-posterior thoracic diameter, transverse thoracic diameter, and thoracic aspect ratio were analyzed. There were no statistically significant differences in height, weight, BMI, intercostal width, or thoracic transverse diameter among the three groups (*p* > 0.05); however, there were significant differences in sternum length, cardiothoracic ratio, anterior-to-posterior thoracic diameter, and thoracic aspect ratio among the three groups (*p* < 0.05; Tables 2 and 3).

**Table 2.** Measurement data of the three groups.

	Deep group	Correct group	Shallow group	*P*
Height (cm)	163.06 ± 9.55	164.14 ± 8.57	162.63 ± 9.62	0.651
Weight (kg)	64.13 ± 12.09	63.63 ± 13.53	63.47 ± 15.00	0.978
Body mass index (kg/m^2^)	23.64 (21.25, 27.30)	23.31 (21.08, 26.53)	23.71 (19.75, 27.07)	0.904
Sternal length (cm)	20.29 ± 1.66	20.99 ± 1.56	18.63 ± 1.68	**<0.001**
Intercostal width (cm)	2.09 ± 0.29	2.10 ± 0.29	2.05 ± 0.30	0.639
Cardiothoracic ratio	0.55 ± 0.05	0.60 ± 0.06	0.61 ± 0.07	**<0.001**
Anterior-to-posterior thoracic diameter (cm)	22.66 ± 2.44	21.45 ± 2.01	20.60 ± 1.32	**<0.001**
Thoracic transverse diameter (cm)	30.31 ± 3.14	30.18 ± 3.22	30.30 ± 3.91	0.972
Thorax aspect ratio	0.78 ± 0.07	0.72 ± 0.06	0.67 ± 0.09	**<0.001**

**Table 3.** Comparison of the measurement data between the three groups.

	Deep group	Correct group	Shallow group	*P*
Sternal length(cm)	20.29 ± 1.66	20.99 ± 1.56	18.63 ± 1.68	0.033 **<0.001**
Cardiothoracic ratio	0.55 ± 0.05	0.60 ± 0.06	0.61 ± 0.07	<0.001 **0.620**
Anterior-to-posterior thoracic diameter(cm)	22.66 ± 2.44	21.45 ± 2.01	20.60 ± 1.32	0.003 **0.041**
Thorax aspect ratio	0.78 ± 0.07	0.72 ± 0.06	0.67 ± 0.09	<0.001 **<0.001**

Note: The *p*-value in normal font indicates a comparison between the Deep group and Correct group; The *p*-value in bold font indicates a comparison between the Shallow group and Correct group.

## Subgroup analysis and exploration of insertion depth

### Comparative analysis of groups 1 and 2

To further explore the factors affecting the excessively deep tip of the catheter, patients were administered corresponding treatments and divided into two groups: group 1, which included the correct group and the shallow group; and group 2, which included the deep group. Statistical analyses were conducted for height, weight, BMI, width of the intercostal space (L2, L3), sternum length, cardiothoracic ratio, anterior-to-posterior thoracic diameter, transverse thoracic diameter, and thoracic aspect ratio. The results showed that there were no statistically significant differences in height, weight, BMI, sternal length, intercostal width, or thoracic transverse diameter between the two groups (*p* > 0.05); however, there were significant differences in the cardiothoracic ratio, anterior-to-posterior thoracic diameter, and thoracic aspect ratio between the two groups (*p* < 0.05; Table 4).

**Table 4.** Comparison between groups 1 (correct and shallow groups) and 2 (deep group) for each measurement index.

	Group 1	Group 2	t/X^2^	*P*
Height (kg)	163.82 ± 8.79	163.06 ± 9.55	0.434	0.665
Weight (cm)	63.59 ± 13.80	64.13 ± 12.09	−0.201	0.841
Body mass index (kg/m^2^)	23.39 (20.20, 25.84)	23.64 (20.65, 26.53)	−0.446	0.656
Sternal length (cm)	20.48 ± 1.85	20.29 ± 1.66	0.539	0.591
Intercostal width (cm)	2.09 ± 0.29	2.09 ± 0.29	0.034	0.973
Cardiothoracic ratio	0.60 ± 0.06	0.55 ± 0.05	−3.983	**<0.001**
Anterior-to-posterior thoracic diameter (cm)	21.27 ± 1.91	22.66 ± 2.44	−3.503	**0.001**
Thoracic transverse diameter (cm)	30.21 ± 3.36	30.31 ± 3.14	−0.164	0.870
Thorax aspect ratio	0.71 ± 0.07	0.78 ± 0.07	−4.628	**<0.001**

### Analysis of factors influencing the depth of the catheter tip

For the univariate logistic regression analysis, the position of the catheter tip was the dependent variable (1 in the deep group and 0 in the correct and shallow groups), and the height, body weight, BMI, sternal length, intercostal width, cardiothoracic ratio, anterior-to-posterior thoracic diameter, transverse thoracic diameter, and thoracic aspect ratio were the independent variables. The results showed that height, body weight, BMI, sternal length, intercostal width, and thoracic transverse diameter had no significant influence on the occurrence of catheter tip position overshoot (*p* > 0.05); however, the cardiothoracic ratio, anterior-to-posterior thoracic diameter, and thoracic aspect ratio influenced the occurrence of catheter tip overshoot (*p* < 0.05; Table 5).

**Table 5.** Univariate logistic regression analysis of factors influencing catheter tip depth.

	B	SE	Wals	*P*	OR	95% CI
Height (kg)	−0.01	0.022	0.191	0.662	0.99	0.948–1.034
Weight (cm)	0.003	0.014	0.041	0.841	1.003	0.975–1.032
Body mass index (kg/m^2^)	0.021	0.046	0.201	0.654	1.021	0.933–1.117
Sternal length (cm)	−0.057	0.106	0.293	0.589	0.944	0.768–1.162
Intercostal width (cm)	−0.023	0.662	0.001	0.973	0.971	0.267–3.581
Cardiothoracic ratio	−0.831	0.417	3.973	**0.046**	0.436	0.192–0.986
Anterior-to-posterior thoracic diameter (cm)	0.316	0.100	10.096	**0.001**	1.372	1.129–1.668
Thoracic transverse diameter (cm)	0.011	0.059	0.027	0.869	1.011	0.901–1.134
Thorax aspect ratio	1.159	0.403	8.280	**0.004**	3.186	1.447–7.013

SE: standard error; OR: odds ratio; CI: confidence interval.

For the multivariate logistic regression analysis, the catheter tip position was used as the dependent variable (1 for the deep group and 0 for the correct and shallow groups), and the variables with statistical significance (*p* < 0.05) in the univariate logistic regression analysis were the independent variables. The results showed that the anterior-to-posterior thoracic diameter was an independent risk factor for catheter tip overshoot (odds ratio [OR]: 1.370; 95% confidence interval [CI]: 1.111–1.690, *p* = 0.003), and a large thoracic aspect ratio was an independent risk factor for catheter tip depth (OR: 2.870; 95% CI: 1.233–6.680, *p* = 0.014). A high cardiothoracic ratio was an independent protective factor against catheter tip overshoot (OR: 0.326; 95% CI: 0.133–0.799, *p* = 0.014; Table 6).

**Table 6.** Multivariate logistic regression analysis of factors influencing the occurrence of deep catheter tip position.

	B	SE	Wals	*P*	OR	95% CI
Cardiothoracic ratio	−1.120	0.457	6.011	**0.014**	0.326	0.133–0.799
Anterior-to-posterior thoracic diameter (cm)	0.315	0.107	8.640	**0.003**	1.370	1.111–1.690
Thorax aspect ratio	1.054	0.431	5.985	**0.014**	2.870	1.233–6.680

SE: standard error; OR: odds ratio; CI: confidence interval.

### Diagnostic value of the depth of the catheter tip using the thoracic aspect ratio and anterior-to-posterior thoracic diameter

**Figure 3.**
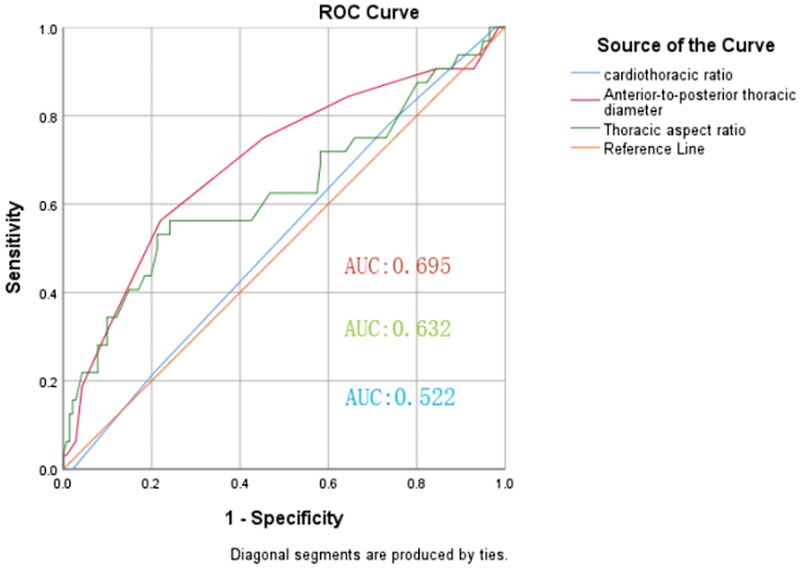
The value of predicting the depth of the catheter position using the anterior-to-posterior thoracic diameter, thoracic aspect ratio and cardiothoracic ratio.

The results of the ROC analysis showed that the feasibility area of the anterior-to-posterior thoracic diameter on the catheter tip was 0.695 (95% CI: 0.5866–0.804, *p* = 0.001), with an optimal cutoff value of 22.5 cm (sensitivity, 56.3%; specificity, 78%), whereas the feasibility area of the horizontal aspect ratio for the catheter tip being too deep was 0.632 (95% CI: 0.513–0.752, *p* = 0.020), with an optimal cutoff value of 0.74 (sensitivity, 56.3%; specificity, 75.9%; Figure 3 and Table 7).

**Table 7.** Value of predicting the depth of catheter position using the anterior-to-posterior thoracic diameter and thoracic aspect ratio.

	Optimum cutoff value	Sensitivity	Specificity	Youden Index	AUC	95%CI	*P*
Anterior-to-posterior thoracic diameter (cm)	22.5	0.563	0.78	0.343	0.695	0.586–0.804	0.001
Thorax aspect ratio	0.74	0.563	0.759	0.322	0.632	0.513–0.752	0.020
Cardiothoracic ratio	0.55	0.781	0.262	0.043	0.522	0.414-0.63	0.696

AUC: area under the curve; CI: confidence interval.

## Discussion

Autogenous arteriovenous fistulas, arteriovenous grafts, and TCCs are the three forms of long-term access that are most commonly used in hemodialysis [2]. However, the TCC plays an important transitional role during the maturation of arteriovenous fistulas, which take up to 1 month or more to develop, or grafts, which usually take approximately 1 month. A considerable number of patients rely on catheters for long-term hemodialysis because of vascular conditions; hence, correct placement of the catheter tip can reduce the occurrence of catheter-related complications [8,9]. According to the consensus of experts on vascular access, the catheter tip should be located at the upper 1/3 of the right atrium, and the third lower intercostal margin of the right margin of the sternum should be used as the target position.

Owing to the limited resources of digital subtraction angiography, when a right internal jugular vein tunnel catheter is placed for the first time in clinical practice, the catheter insertion depth is usually estimated based on the measurement of the puncture location and body surface markers. However, the direction of the catheter does not completely follow the preset path on the body surface; therefore, the measurement length is often biased, and the catheter tip cannot be placed at once, which requires adjustment of the catheter position. This often leads to an increased incidence of dialysis catheter infections. The deviation that occurs may be due to the thoracic thickness, anterior chest wall muscles, and subcutaneous adipose tissue, which are not parallel to the superior vena cava. The thickness of the thorax can be expressed by its anterior-to-posterior and transverse diameters. The larger the ratio of the anterior-to-posterior thoracic diameter to the transverse thoracic diameter (thoracic aspect ratio), the thicker the thorax, and the greater the difference between the surface positioning of the catheter tip and the actual depth *in vivo*. A total of 173 patients were included in this study, and among them, 36% had a catheter tip position that was too deep or shallow. Therefore, the clinical use of the third inferior intercostal margin as a body surface marker to predict the position of the catheter tip leads to the positioning being too deep or too shallow. In addition, clinicians have noted that there is a certain degree of error in the positioning of the surface intercostal space. In most patients, the position of the catheter tip has a range of motion of 2–3 cm in the body, which is affected by breathing and body position [10,11]. Previous studies have shown that, in the absence of fluoroscopy, the appropriate catheter length position can be estimated through ultrasound guidance and measurements of the patient’s height and recent chest X-ray. Alternatively, the manubrial–sternal angle can be used as a marker to estimate the insertion depth based on the distance between the puncture site and the point 5 cm below the manubrial–sternal angle. Both surface positioning methods can achieve successful insertion of the tunnel catheter, with no difference in the incidence of catheter dysfunction [5].

We found that the third intercostal space of the right margin of the sternum was used as the catheter method for body surface projection of the catheter tip, and the position of the catheter tip was related to the sternum length and cardiothoracic ratio of the patients. The sternum is clearly visible on the sternal surface, the xiphoid process is easily recognized, and the manubrium joins the body in the horizontal plane, passing through the carina and right atrium, making the length of the sternum easily measurable [12]. The cardiothoracic ratio is the maximum horizontal diameter of the heart divided by the horizontal width inside the chest, expressed as a ratio; the normal value is <0.5. When the cardiothoracic ratio is >0.5, the heart shadow of the patient is enlarged. In this study, patients with end-stage renal disease were enrolled, and their cardiothoracic size was greater than normal. Studies have suggested that patients with end-stage renal disease have larger hearts than healthy people do, and with increasing dialysis time, the cardiothoracic ratio also changes to a certain extent [13]. In this study, the position of the TCC catheter tip was related to the sternal length. When comparing the deep group, shallow group, and normal group, there was a statistically significant difference in sternal length, indicating that patients in the shallow group had shorter sternal length. To examine factors influencing excessive insertion, we combined data from the shallow and normal groups and compared them with those in the deep group; we found that there was no statistically significant difference in sternum length. The reason is that the difference in average sternum length data between the normal group and the shallow group was smaller than that between the normal group and the deep group. Fereshte Salimi et al. [14] noted that patients under the age of 55, with a height greater than 155 cm and a sternum length of 21.5 cm or less, should apply the current body surface labeling method for hemodialysis catheter placement, which would be suitable for their stature. Therefore, in clinical catheterization, attention should be given to the measurement of sternum length in each patient.

We also found that the anterior-to-posterior thoracic diameter and the thoracic aspect ratio were related to the position of the catheter tip. Relevant studies have indicated that the thoracic aspect ratio is correlated with the depth of catheter implantation. The reason for this difference may be that the thoracic aspect ratio affects the anatomical position of the right atrial junction (cavoatrial junction); where the anterior-to-posterior thoracic diameter is decreased, the transverse thoracic diameter is widened or unchanged, and the thoracic aspect ratio is reduced, which changes the structure and position of the heart and leads to changes in the position of the cavoatrial junction. The implantation depth also changes [15]. In this study, height was not found to be related to the position of the catheter tip, considering that the length of catheter insertion required for each person was different and that there was no obvious correlation between height and catheter insertion length. However, height has also been included in some studies on catheter implantation depth, and several formulas for predicting catheter insertion depth based on height have been proposed [16]. In clinical practice, we should not only consider the height of patients but also pay attention to the impact of BMI on the predicted length of the catheter. For patients with stocky physiques, the body surface reference point should be moved upward during the operation; otherwise, the catheter tip position will easily become too deep.

Factors related to catheter tip position, such as sternal length, cardiothoracic ratio, anterior-to-posterior thoracic diameter, and thoracic aspect ratio, were identified. In the multivariate logistic regression analysis, the anterior-to-posterior thoracic diameter length and a large thoracic aspect ratio were found to be independent risk factors for catheter tip position overshoot. Therefore, we recommend that the body surface positioning be moved upward in such patients. The ROC curve showed that the length of the anterior-to-posterior thoracic diameter and a large thoracic aspect ratio had predictive value for overshooting; the areas under the curve were 0.695 and 0.632, respectively, and the optimal cutoff values were 22.5 cm and 0.74, respectively. When the current catheterization method is used, the position of the catheter tip is too deep if following the cutoff values. Univariate logistic regression analysis revealed that the cardiothoracic ratio influenced the excessively deep position of the catheter tip; however, the optimal cutoff value was not obtained when the ROC curve was drawn, which may be related to certain changes in the cardiothoracic ratio and cardiac structure with the increase in dialysis time.

Clinicians should should carefully consider sternal length, cardiothoracic ratio, anterior-to-posterior thoracic diameter, and thoracic aspect ratio of patients during catheterization to reduce the occurrence of catheter-related complications and providing patients with stable and reliable long-term vascular access. Indices such as sternum length, cardiothoracic ratio, anterior-to-posterior thoracic diameter, thoracic transverse diameter, and thoracic aspect ratio were included to predict whether the catheter tip position was correct. Factors related to the catheter tip position were also found to increase the accuracy of the catheter tip position and make the procedure more individualized.

However, there were several limitations in this study. First, only patients with right internal jugular vein catheters were included in this study, and the patients were only from our hospital, resulting in a small sample size. In the future, multicenter studies with larger sample sizes are required for further discussion. Second, this was a cross-sectional observational study, and no analysis was conducted after adjusting for inappropriate catheters. Further research is required to determine how the position of the catheter tip should be adjusted. Third, we only observed catheters with a stepped tip. Symmetrical and split-tip catheters are associated with a lower risk of catheter dysfunction than step-tip catheters [17]. Multifaceted interventions that promote evidence-based catheter care may prevent dysfunction, mainly because the intervention may have influenced catheter management practices that ultimately improved catheter tip position or prevented thrombosis[18]. Therefore, we believe that for any type of catheter, only the functional segment of the catheter should be placed in the appropriate position, which can provide good blood flow and reduce the risk of complications. Fourth, we used three-dimensional CT reconstruction to determine the positional relationship between the catheter tip and the atrium; although three-dimensional CT reconstruction is more intuitive than regular chest X-rays, it increases the level of radiation exposure of patients.

## Conclusion

The third lower intercostal margin of the right margin of the sternum was used as the positioning marker for the body surface projection of the catheter tip. We found that the position of the TCC catheter tip was related to the sternal length, anterior-to-posterior thoracic diameter, thoracic aspect ratio, and cardiothoracic ratio. In addition, the length of the anterior-to-posterior thoracic diameter and a large thoracic aspect ratio were found to be independent risk factors for overshooting the catheter tip.

## Data Availability

Data will be shared upon reasonable request from the corresponding author.
